# The diffusion model’s drift rate parameter primarily reflects efficiency, rather than speed, of evidence accumulation

**DOI:** 10.3758/s13423-026-02861-3

**Published:** 2026-02-26

**Authors:** Alexander Weigard, M. Fiona Molloy, Chandra Sripada, Andrew Heathcote

**Affiliations:** 1 Department of Psychiatry, University of Michigan, Rachel Upjohn Building, 4250 Plymouth Road, Ann Arbor, MI 48109 USA; 2 University of Michigan Weinberg Institute for Cognitive Science, Ann Arbor, MI USA; 3 Department of Philosophy, University of Michigan, Ann Arbor, MI USA; 4 Department of Psychological Methods, University of Amsterdam, Amsterdam, The Netherlands

**Keywords:** Processing speed, Mathematical models, Individual differences, Cognitive ontologies

## Abstract

Applications of the diffusion decision model (DDM) to the study of cognitive individual differences consistently find that the model’s drift rate (*v*) parameter forms a cohesive factor across many tasks and relates to measures of higher-order cognitive functioning, including general cognitive ability and working memory. This parameter is often interpreted as a measure of “processing speed,” a traditional psychometric construct thought to reflect an individual’s basic speed of information processing across tasks. However, conceptual differences between* v* and traditional notions of processing speed make this mapping far from straightforward. Racing accumulator models, which provide a more flexible and comprehensive account of behavioral data than the DDM, allow for the *speed* with which individuals accumulate evidence to be dissociated from the *efficiency* with which they accumulate task-relevant evidence (versus task-irrelevant evidence). We applied the DDM and a racing accumulator model to three tasks across three independent datasets to gauge the extent to which *v* parameter findings from the cognitive individual differences literature reflect speed of evidence accumulation (SEA) versus efficiency of evidence accumulation (EEA). Across all tasks, *v* was more strongly related to EEA than SEA. EEA was consistently related to measures of general cognitive ability, working memory, and executive function whereas SEA explained <1% of the variance in each. These findings suggest individual differences in the DDM’s *v* parameter, and its relations with higher-order cognitive abilities, primarily reflect EEA rather than SEA and challenge the widespread practice of equating *v* with the traditional “processing speed” construct.

**Supplementary Information:**

The online version contains supplementary material available at 10.3758/s13423-026-02861-3.

## Introduction

**Fig. 1:**
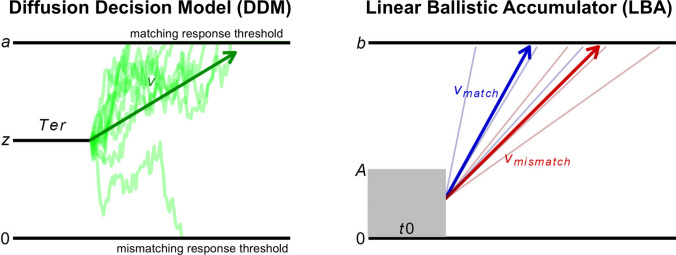
Schematics of the diffusion decision model (DDM) and a commonly used accumulator model, the linear ballistic accumulator (LBA). In the DDM, noisy evidence accumulation causes a single evidence total to drift over time from a start point (*z*) between two boundaries alternately representing the response that matches the stimulus (set at ***a***) and the response that mismatches the stimulus (set at 0). A single drift rate (*v*) parameter determines the average rate at which the evidence accumulation process drifts towards the matching response boundary. In the LBA, linear ballistic accumulators for responses matching, versus mismatching, the stimulus start at a point drawn from a uniform distribution (bounded by 0 and *A*) and race towards a common response threshold (***b***). The accumulators have rates that vary across trials (as opposed to the within-trial variability assumed by the DDM) and their mean rates are described by *v*_*match*_ and *v*_*mismatch*_ parameters. Both models also account for “nondecision” time spent on peripheral perceptual and motor processes (*Ter* in the DDM, *t0* in the LBA). In the LBA, efficiency of evidence accumulation (EEA) can be measured by subtracting *v*_*mismatch*_ from *v*_*match*_ and speed of evidence accumulation (SEA) can be measured as the mean (or sum) of *v*_*match*_ and *v*_*mismatch*_. (Color figure online)

Cognitive models posit formal, mathematically specified explanations for how people complete cognitive tasks. Given the longstanding interest in cognitive science with understanding reasons for individual differences in cognitive performance (Ackerman & Lohman, 2006; Carroll, 2003; Kane & Engle, 2002), fitting these models to individuals’ empirical data provides a unique opportunity to characterize mechanistic processes that drive such differences (Lerche et al., 2020; Ratcliff et al., 2011; Schubert & Frischkorn, 2020). The diffusion decision model [DDM]; Ratcliff, 1978; Ratcliff et al., 2016) posits that individuals generate responses on cognitive tasks requiring binary decisions by accumulating noisy evidence from stimuli until the accumulation process crosses an evidence threshold for one response (Fig. 1). The DDM is highly influential due to its success in providing empirically well-supported, psychologically grounded explanations of a wide variety of behavioral phenomena (Dutilh et al., 2019; Ratcliff et al., 2016; Starns et al., 2019; Voss et al., 2004).

One of the most consistent findings from this literature concerns the DDM’s drift rate (*v*) parameter, which indexes the average rate at which the evidence accumulation process approaches the threshold for the correct response (Fig. 1). Although *v* was originally characterized as reflecting the quality of stimulus information in the experimental literature from which the DDM emerged (Leite & Ratcliff, 2011), individual differences studies have repeatedly demonstrated that* v* reliably forms a cohesive and trait-like general factor that drives individual differences in performance across many tasks (Eisenberg et al., 2019; Lerche et al., 2020; Löffler et al., 2024; Schmiedek et al., 2007; Schubert et al., 2016; Weigard et al., 2021). Further, individual differences in the *v* parameter, even measured on relatively simple two-choice perceptual decision-making paradigms, appear to be closely related to measures of individuals’ higher-order cognitive functioning, including general cognitive ability (also called “general intelligence”: Carroll, 2003; McGrew, 2009), working memory, and executive functions (Karalunas & Huang-Pollock, 2013; Lerche et al., 2020; Löffler et al., 2024; Schmiedek et al., 2007; Schmitz & Wilhelm, 2016; Schubert & Frischkorn, 2020; Weigard, Suzuki, et al., 2024b; Weigard & Huang-Pollock, 2017). Hence, *v* presents as a compelling candidate for a basic cognitive capacity that influences individuals’ ability to accurately complete a wide variety of complex cognitive operations.

It has been commonplace throughout the individual differences literature to refer to the DDM’s* v* parameter as an index of “processing speed” or “speed of information processing” (Hedge et al., 2022; Karalunas & Huang-Pollock, 2013; Lerche et al., 2020; Löffler et al., 2024; Ratcliff et al., 2011, 2012; Reinhartz et al., 2023; Schubert et al., 2015; Schubert & Frischkorn, 2020; van Ravenzwaaij et al., 2011; Vermeent, Young, DeJoseph, et al., 2024a, Vermeent, Young, Van Gelder, et al., 2024b), including in prior work by the first author of this report (Weigard & Huang-Pollock, 2017). According to the traditional conceptualization of “processing speed” from the psychometric literature, this capacity represents the speed with which individuals process information across essentially all cognitive tasks (Fry & Hale, 1996, 2000; Kail & Salthouse, 1994; Salthouse, 1996). As processing speed is thought to be an elemental ability, it is typically measured as the average response time (RT) on relatively simple cognitive tasks that, in theory, have few higher-order demands on information processing (e.g., top-down control demands).^1^ Because the DDM’s *v* parameter has some conceptual similarities to this theoretical construct, and because it shows evidence of being a domain-general process that influences performance on even simple tasks, linking the two is understandable.

However, this link is not entirely straightforward. Although the effects of processing speed on performance have been assumed to manifest primarily in the latencies of RTs, the *v* parameter strongly affects the variability and positive skew of RT distributions as well as their mean latency (Matzke & Wagenmakers, 2009). Further, mean RT latency is also largely explained by alternative DDM parameters for perceptual encoding, motor speed, and response caution (Matzke & Wagenmakers, 2009). Therefore, a positive manifold in mean RTs (Thurstone, 1931) is likely to be explained, in part or in whole, by alternative processes such as an individuals’ overall level of response caution (Hedge et al., 2019; Weigard et al., 2021), rather than exclusively by *v*. This substantial theoretical and methodological disconnect between the *v* parameter and the construct of processing speed—as the latter construct has been defined and measured in decades of previous literature—indicates that that equating the two constructs is likely to create confusion.

Perhaps more importantly, the *v* parameter does not actually measure the overall rate at which a person accumulates information, but rather the rate at which the information they accumulate moves the decision process towards the threshold for the *correct response*. An individual may be able to quickly accumulate information about a stimulus, but if they are inefficient at parsing information relevant to the task goal from task-irrelevant information, the quality of the accumulated evidence and, therefore, the value of their *v* parameter will nonetheless be low.

The more flexible class of racing accumulator models, which have a similar track record to the DDM of successfully explaining patterns in behavioral data (Brown & Heathcote, 2008; Heathcote & Matzke, 2022; Usher & McClelland, 2001), provide a way of disentangling the overall speed of evidence accumulation from an individual’s ability to accumulate task-relevant over irrelevant evidence. These models assume that noisy evidence for each response is gathered in separate accumulators that race, with the first to reach its threshold triggering the corresponding response (Fig. 1). In this framework, two separate dynamics govern the evidence accumulation process:

1. The overall *speed* of evidence accumulation (SEA), captured by the average or sum of the accumulation rates, and
2. The *efficiency* of evidence accumulation (EEA), captured by the difference between the rate of the accumulator matching the correct response and the rate(s) of mismatching accumulators.

As with the DDM’s *v* parameter, neither EEA nor SEA has an exact mapping to the traditional theoretical construct of processing speed. However, SEA is much more closely theoretically aligned with this construct because it reflects individuals’ overall speed of accumulating evidence, regardless of the quality of that evidence. In contrast, EEA represents individuals’ ability to efficiently parse task-relevant information from irrelevant information, which is orthogonal to SEA and has little conceptual overlap with the theoretical processing speed construct. At the level of how the theoretical processing speed construct has been empirically defined—as mean choice RT—SEA almost exclusively affects mean choice RT while EEA primarily affects accuracy, as detailed in simulations outlined in Supplemental Materials.^2^ Hence, traditional measures of “speed” may be influenced by either EEA or SEA, but SEA has a more selective relationship. One prior study applying accumulator models to a battery of decision-making tasks has identified a positive manifold in EEA that mimics the positive manifold in the DDM’s *v* parameter (Stevenson et al., 2024). Yet despite the widespread practice of referring to the DDM’s *v* parameter as “speed,” no prior studies have explicitly used racing accumulator models to compare EEA and SEA’s empirical relations with the DDM’s *v* parameter. Nor have prior studies clarified whether EEA or SEA plays a similar role to *v* in supporting higher-order cognitive processes.

The current report sought to fill this gap by simultaneously estimating parameters of the DDM and the linear ballistic accumulator (LBA) model (Brown & Heathcote, 2008) in three independent samples. We evaluated how closely EEA and SEA estimates were related to the DDM’s *v* parameter and assessed whether EEA, SEA, or both processes played a similar role to *v* in supporting performance on tasks that involve complex cognitive processing.

## Methods

### HCP sample and task

The Human Connectome Project (HCP) is consortium study that aimed to use functional neuroimaging to facilitate the mapping of the human connectome by collecting and freely distributing data from 1,200 healthy young adults, aged 22–35 (Van Essen et al., 2013). The HCP consortium recruited a sample of twins and their nontwin siblings from the local communities of consortium sites to facilitate estimation of heritability in connectome features. Participants completed an extensive battery of both task-based and resting state functional neuroimaging measures and behavioral testing. Data for the HCP sample were taken from the HCP-1200 release (Van Essen et al., 2013; WU-Minn, 2017).

We used data from the HCP’s n-back task, which was completed during functional neuroimaging data acquisition. In each of two conditions of this task, participants were presented with a series of images of faces, places, tools and body parts in 10-trial blocks. In the 0-back condition, participants were shown a target image during a 2.5-s cue at the beginning of each block and were asked to respond as to whether each image presented matched the target image. In the 2-back, participants were asked to evaluate whether the presented stimulus was the same as the stimulus presented two trials back. Therefore, the 0-back was essentially a simple recognition memory task while the 2-back required the active maintenance of information in working memory. In addition to “target” trials, both task conditions included “lure” trials, which were stimuli that were presented before in the block but that did not meet target criteria, and “novel” trials that were never presented before.

Of the 1,073 individuals in the HCP-1200 release with complete n-back task data, 0-back data from all 1,073 individuals and 2-back data from 1,065 individuals met data quality inclusion criteria (outlined below). In addition, the LBA model displayed persistent convergence difficulties with one participant’s 0-back data, leaving total samples of 1,072 for the 0-back and 1,065 for the 2-back.

### ABCD sample and task

The Adolescent Brain Cognitive Development Study (ABCD)^®^ is a large, multi-site longitudinal study of 11,875 U.S. youth recruited at ages 9–10 at 22 consortium sites (Casey et al., 2018). Youth were recruited using school-based sampling methods designed to allow for a sample that was reflective as possible of the diversity of the U.S. population (Garavan et al., 2018). Twins and nontwin siblings were recruited, similar to HCP, but most participants were from different families. We used data from the ABCD baseline session (ages 9–10) included in ABCD Release 5.0.

ABCD study participants completed an n-back task with 0-back and 2-back blocks during neuroimaging data acquisition that each had an identical design to the corresponding tasks used in HCP, except for the stimuli used. The ABCD 0-back and 2-back included blocks that presented face stimuli with happy, fearful or neutral facial expressions as well as blocks that presented images of places, a design choice intended to elicit activity in “emotion” and “place” processing brain regions.

Of the 10,042 individuals with complete *n*-back data at the ABCD baseline session, 0-back data from 9,286 individuals and 2-back data from 9,104 individuals met data quality inclusion criteria (outlined below). In addition, the LBA model displayed persistent convergence difficulties with two participants’ 0-back data, leaving total samples of 9,284 for the 0-back and 9,104 for the 2-back.

### Prolific numerosity discrimination sample and task

The final dataset was drawn from an online study that included Simon and flanker conflict tasks and a numerosity discrimination task (Molloy et al., in press), the last of which was included in the current report due to it being a basic perceptual discrimination task that is commonly used in the DDM literature (Leite & Ratcliff, 2011; Ratcliff, 2008; Ratcliff et al., 2012; Weigard & Huang-Pollock, 2017). Participants recruited using the Prolific online research platform (*n* = 234) were presented with arrays of asterisks on a 10 × 10 grid and asked to report if there were “few” (<50) or “many” (>50) stimuli. The task contained both two difficulty conditions, hard (41–45 or 55–59 asterisks) and easy (31–35 or 65–69 asterisks), with 100 trials each (200 total) and trials were randomly interspersed. Of the 234 participants recruited, numerosity discrimination data from 226 individuals (mean age = 27.64 years, age range: 18–35 years, 115 women) met data quality inclusion criteria outlined below.

### Criterion measures

Both the ABCD and HCP data sets contained the NIH Toolbox Battery, which was designed to be a comprehensive and efficient battery capable of measuring an array of cognitive domains across youth and adult participants (Akshoomoff et al., 2013; Weintraub et al., 2013). The NIH Toolbox Total Composite Score, which reflects performance across all domains in the NIH Toolbox battery, was used as a measure of general cognitive ability. The List Sorting Working Memory test, which involves the serial presentation of pictures and names of animals or foods of different sizes followed by a request for the participant to repeat back the items presented in the order of smallest to largest size, was used as a measure of working memory. The Dimensional Change Card Sort test, which asks participants to sort objects according to two different dimensional criteria (color and shape) that alternate across blocks and are interleaved pseudorandomly in a third block, was used as a measure of cognitive flexibility. The flanker test, a variant of the traditional arrow flanker, was used as a measure of inhibition. Standardized NIH Toolbox scores, uncorrected for age, were used for each of these measures.

### Model estimation and model fit

The DDMs for the 0-back and 2-back tasks allowed drift rate to vary by *n*-back trial type (*v*_*novel,*_* v*_*target*_, *v*_*lure*_), and also included parameters for boundary separation (*a*), start point (*z*), nondecision time and its between-trial variability (*t0*, *st0)*, as well as a “go failure” (*p*_*gf*_) parameter to account for omissions (Damaso et al., 2021). The LBAs for the 0-back and 2-back tasks allowed accumulator drift rates to vary across both trial-type and whether the accumulator’s response was the correct match for the trial stimulus (*v*_*novel-match,*_* v*_*target-match*_, *v*_*lure-match*_,* v*_*novel-mismatch,*_* v*_*target-mismatch*_, *v*_*lure-mismatch*_), and also included parameters for response threshold (*B*), start point variability (*A*), nondecision time (*t0*), “go failure” (*p*_*gf*_), and between-trial variability in the rate of the accumulator mismatching the correct response (*sv*_*mismatch*_). Between-trial variability in the rate of the accumulator matching the correct response (*sv*_*match*_) was fixed to 1 as a scaling parameter (Donkin et al., 2009).

The DDMs for the numerosity discrimination task allowed drift rate to vary by stimulus type and difficulty (*v*_*many-easy,*_* v*_*few-easy*_, *v*_*many-hard*_, *v*_*few-hard*_) and contained the other standard DDM parameters also estimated for the n-back tasks (*a*, *z*, *t0*, *st0, p*_*gf*_). The LBAs for the numerosity discrimination task allowed accumulator drift rates to vary across stimulus type, difficulty, and whether the accumulator’s response was the correct match for the trial stimulus (*v*_*many-easy-match,*_* v*_*few-easy-match*_, *v*_*many-hard-match*_, *v*_*few-hard-match*_, *v*_*many-easy-mismatch,*_* v*_*few-easy- mismatch*_, *v*_*many-hard- mismatch*_, *v*_*few-hard- mismatch*_). We also allowed thresholds to vary by response type (*B*_*many,*_* B*_*few*_) given prior work demonstrating that doing so improves model fit (Weigard et al., 2018) and otherwise estimated the remaining parameters across all trials (*A*, *t0, p*_*gf*_).

Before estimation of any models, participants were excluded for poor data quality if their accuracy rate was below 55% or if they displayed omissions (non-responses) on more than 25% of trials (proportions excluded are reported above). DDM and LBA parameters were then estimated in the Dynamic Models of Choice (DMC) R suite (Heathcote et al., 2019) using individual Bayesian estimation with the “RUN.dmc()” function. Broad and uninformative priors were used for the estimation of both models (Supplemental Tables 1–2). Following prior work (Damaso et al., 2021), both models accounted for the probability that some responses would be omitted due to falling after the response window (2 s for the ABCD and HCP *n*-back tasks, 3 s for the numerosity task) and also estimated the probability of additional omitted responses due to inattention to the task (the “go failure” parameter: *p*_*gf*_). Sampling from parameter posteriors was conducted with the differential evolution Markov chain Monte Carlo (DE-MCMC) method (Turner et al., 2013) and convergence was defined as the Gelman-Rubin statistic falling below 1.10 (Gelman & Rubin, 1992).

**Fig. 2:**
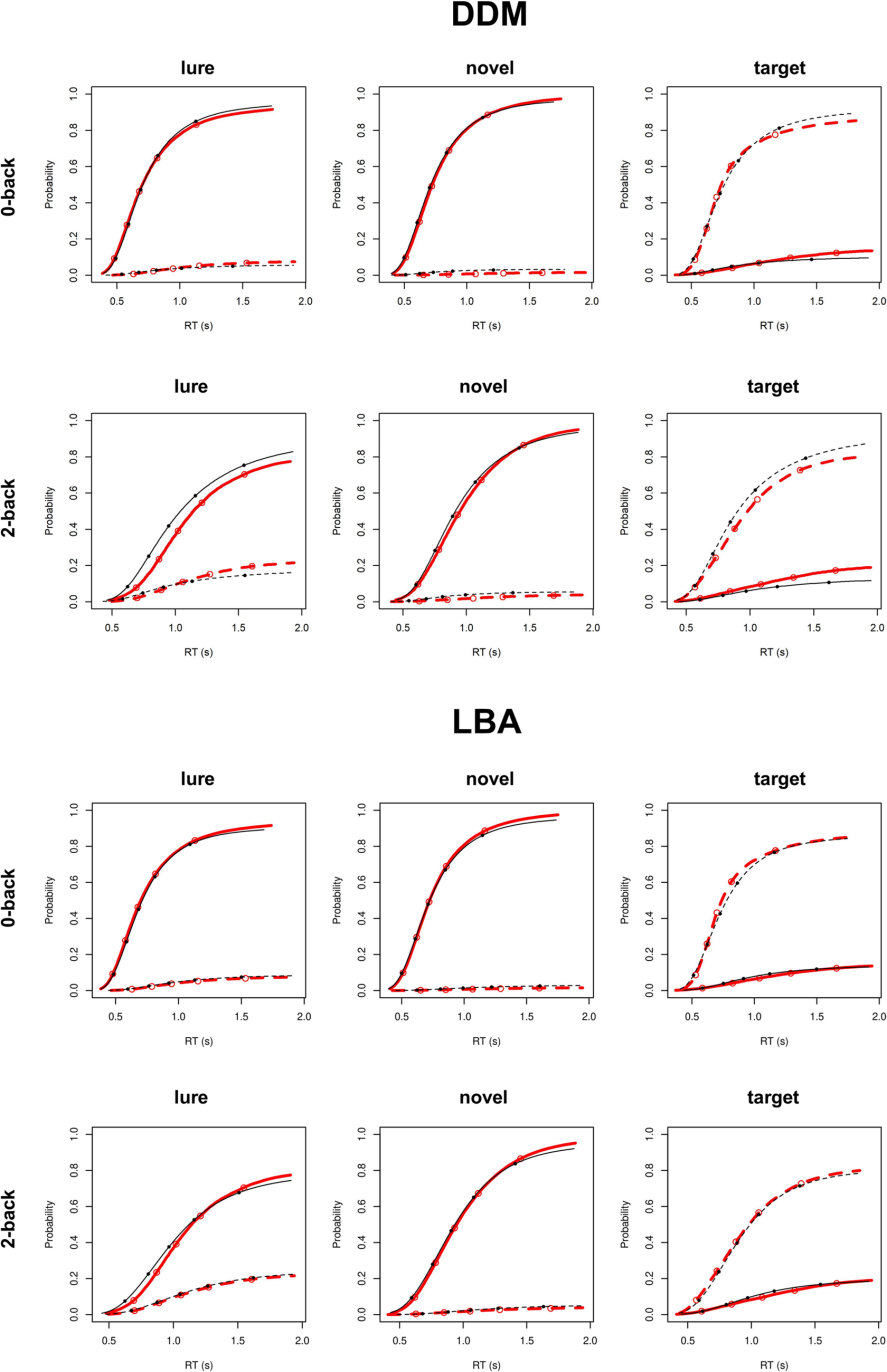
Posterior predictive plots for linear ballistic accumulator (LBA) model and diffusion decision model (DDM) fits to the Human Connectome Project (HCP) *n*-back task data. Each plot displays the cumulative probability of a “target” response (**dotted line**) and a “nontarget” response (**solid line**) for the empirical (red) and model-predicted (black) data. The five points shown in each plot represent response time (RT) quintiles (.1,.3,.5,.7,.9) for the empirical (open point) and model-predicted (solid point) data. (Color figure online)

**Fig. 3:**
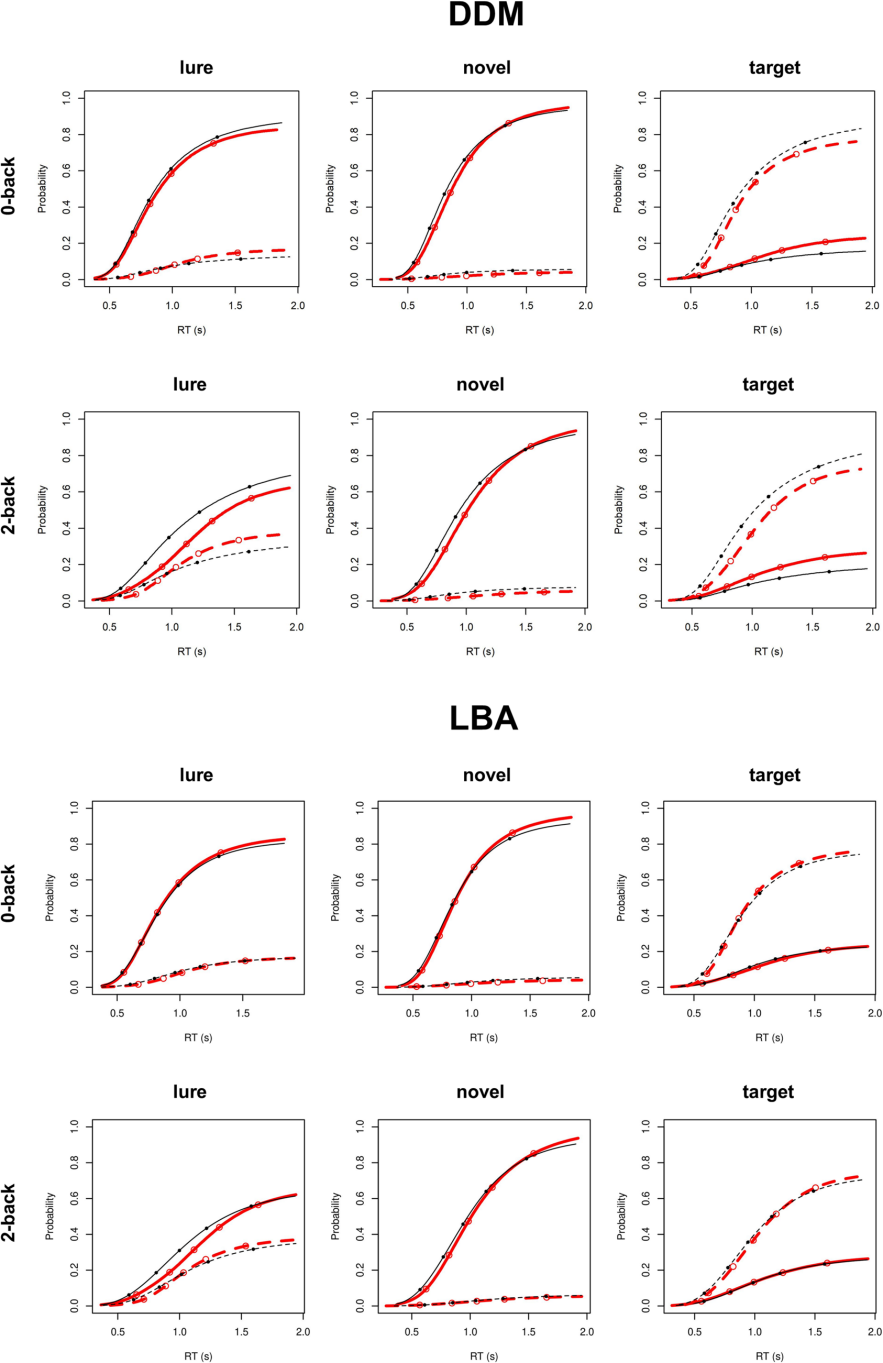
Posterior predictive plots for linear ballistic accumulator (LBA) model and diffusion decision model (DDM) fits to the Adolescent Brain Cognitive Development Study (ABCD) *n*-back task data. Each plot displays the cumulative probability of a “target” response (**dotted line**) and a “nontarget” response (**solid line**) for the empirical (red) and model-predicted (black) data. The five points shown in each plot represent response time (RT) quintiles (.1,.3,.5,.7,.9) for the empirical (open point) and model-predicted (solid point) data. (Color figure online)

**Fig. 4:**
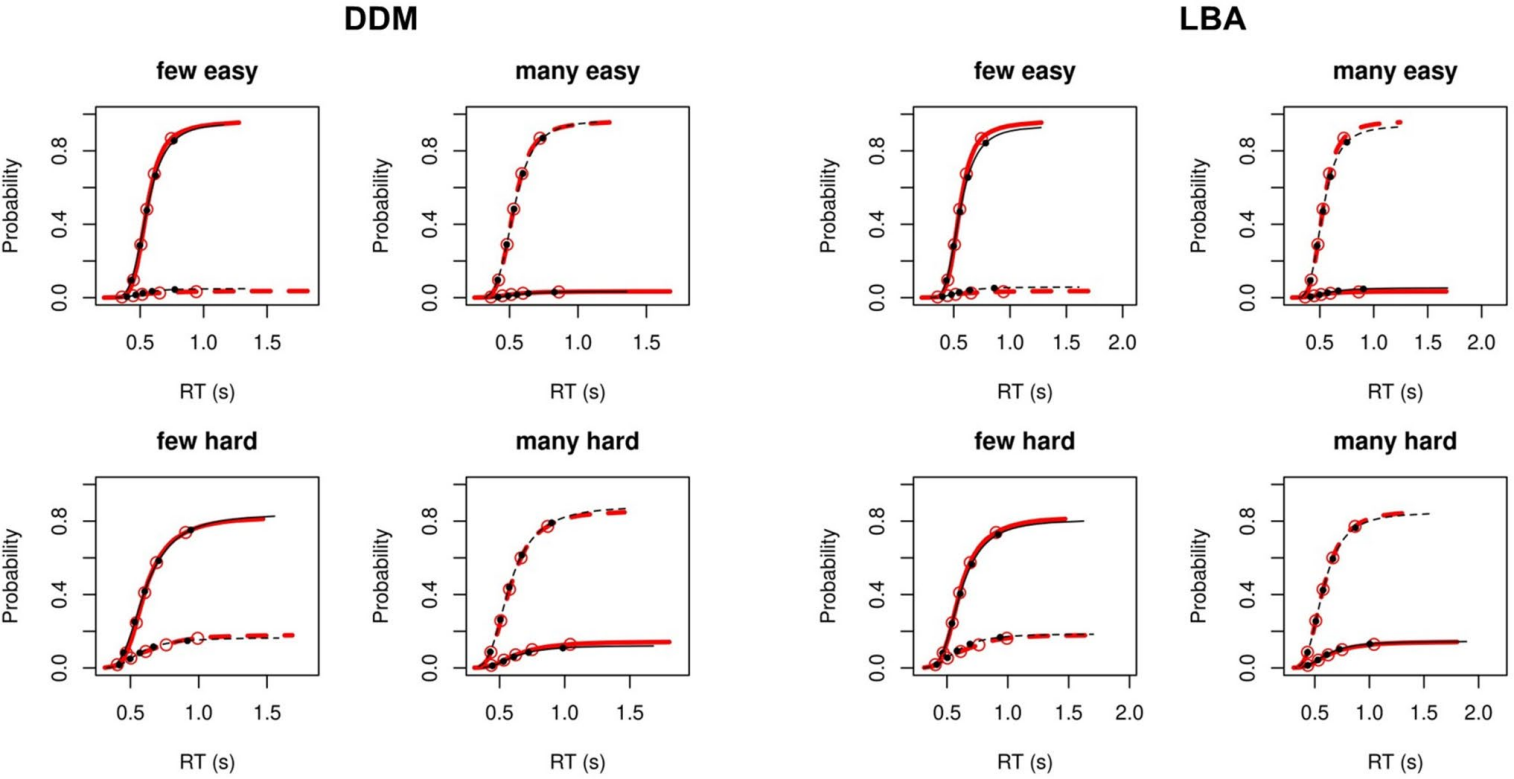
Posterior predictive plots for diffusion decision model (DDM) and linear ballistic accumulator (LBA) fits to numerosity discrimination task data. Each plot displays the cumulative probability of a “many” response (**dotted line**) and a “few” response (**solid line**) for the empirical (red) and model-predicted (black) data. The five points shown in each plot represent response time (RT) quintiles (.1,.3,.5,.7,.9) for the empirical (open point) and model-predicted (solid point) data. (Color figure online)

Model fit was investigated using posterior predictive plots (Gelman et al., 1996) that compare data predicted by the model to the empirical task data. Plots for the HCP, ABCD, and Prolific numerosity discrimination samples are displayed in Figs. 2, 3 and 4, respectively. The LBA displayed very good fit across all tasks and samples as it was generally able to reproduce both choice proportions and RT quantiles across each task condition. The LBA only displayed some minor misfits to error RT quantiles, which tend to be more difficult to estimate due to the lower number of errors than correct trials. The DDM displayed similarly good fit to the 0-back and numerosity discrimination tasks, but displayed greater misfit to the target and lure trial conditions in the 2-back task, overestimating individuals’ accuracy rates in these conditions in both the HCP (Fig. 2) and ABCD (Fig. 3) samples. Notably, previous work using informative priors derived from hierarchical model fits to independent empirical data (Weigard, Angstadt, et al., 2024a; Weigard, Suzuki, et al., 2024b) finds the DDM displays good fit to target and lure trials in these data sets when the model is constrained by the informative priors. Therefore, we attribute these accuracy misfits to our choice to use broad and uninformative priors in this study. Regardless, as the LBA displayed consistently good fit across all tasks, indicating that EEA and SEA estimates are likely to be trustworthy, we investigated whether the DDM’s *v* parameter was reliably related to EEA or SEA across both tasks to which the DDM displayed good fit (0-back, numerosity) and to which it displayed relatively poorer fit (2-back).

EEA was defined by taking the average, across all task conditions, of the difference between drift rates for accumulators that match, versus those that mismatch, the correct response. SEA was defined by taking the average, across all task conditions, of the mean of drift rates for the matching and mismatching accumulators. The DDM’s *v* parameter was similarly averaged across all task conditions.

### Data visualization and inferential analyses

All data visualization and analysis was conducted within R (R Core Team, 2013) and code is available at OSF (osf.io/9nd4h.). Bivariate relations of EEA and SEA with *v* were visualized using the “geom_pointdensity()” function from the *ggplot2* R package. As the ABCD and HCP samples contain data nested by family and consortium site, linear mixed effects models with random intercepts for levels of nested variables were fit using the “lmer()” function from the *lme4* R package to determine the individual contributions of EEA and SEA to explaining variance in DDM* v* and in the cognitive criterion measures. Marginal *r*-squared and bootstrapped (1000 iterations) confidence intervals (CIs) for *r*-squared values were computed using the *partR2* R package.

## Results

### The DDM’s* v* parameter is more closely related to EEA than to SEA

**Fig. 5:**
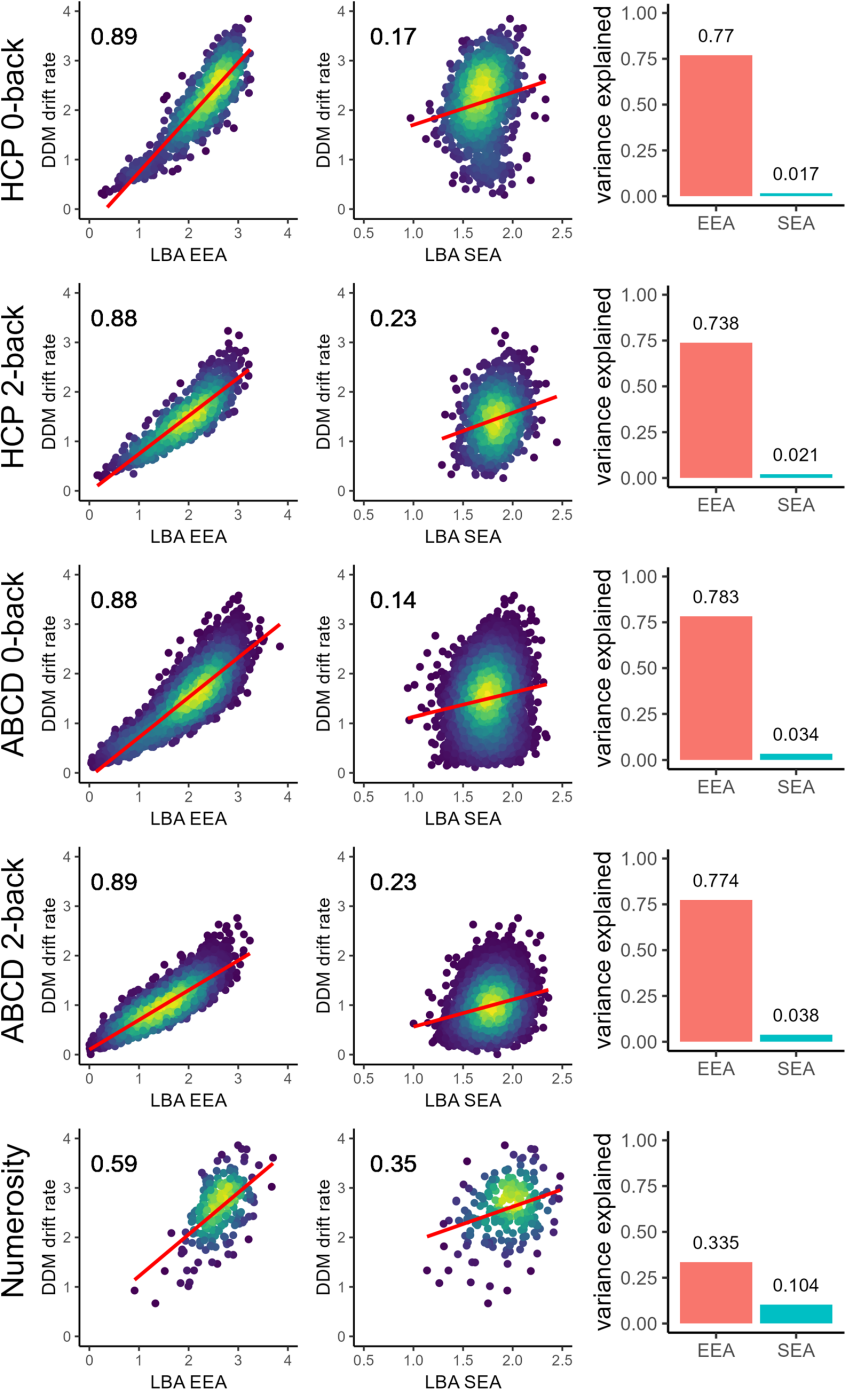
Relations of individuals’ speed of evidence accumulation (SEA) and efficiency of evidence accumulation (EEA), estimated from the linear ballistic accumulator model (LBA), with the drift rate parameter of the diffusion decision model (DDM). Rows represent individual cognitive tasks drawn from the Human Connectome Project (HCP), Adolescent Brain Cognitive Development study (ABCD), and a sample of Prolific participants who completed a numerosity discrimination task online. Scatterplots illustrate the raw correlations of EEA and SEA with drift rate. Pearson correlation estimates are reported in the upper left corner. The color scale reflects the density of the points, with blue indicating lower density and yellow indicting greater density, and the red lines indicate the linear relations between the variables. Bar plots in the rightmost column show the proportion of variance explained in drift rate by EEA and SEA when both parameters are included within the same linear mixed model. (Color figure online)

Relations of EEA and SEA with the DDM’s *v* parameter in all tasks and samples are displayed in Fig. 5. Raw correlations, reported in the scatterplots, indicated that EEA consistently displayed strong relations with *v* (*r* =.59–.89). For the ABCD and HCP tasks the correlations were uniformly above 0.80, suggesting that *v* could be viewed functionally as a proxy measure for EEA in these tasks. In contrast, correlations between SEA and *v* were substantially lower across all tasks (*r* =.14–.35). Multivariate linear models that evaluated the unique contributions of EEA and SEA to explaining variance in *v* indicated that EEA consistently explained a substantially larger proportion of the variance in *v* than SEA did (bar plots in Fig. 5). The strength and selectivity of EEA’s relation to *v* was lowest in the numerosity task data set. However, even in this data set, EEA explained over three times more variance in *v* than SEA did.

We also investigated relations between other LBA and DDM parameters that have similar mechanistic interpretations across the models. These included relations between the height of LBA response thresholds and the DDM boundary separation parameter, both of which are thought to index an individual’s level of caution in responding, and relations between nondecision time parameters across each model. Consistent with prior work (Donkin et al., 2011), correlations of these parameters across the LBA and DDM models (Supplemental Fig. 2) were uniformly strong and positive (*r* >.50), suggesting that the models also provide generally similar accounts of other mechanistic processes.

### Higher-order cognitive functions previously linked to *v* are related to EEA but not SEA

Results from multivariate linear models predicting criterion measures of higher-order cognitive functioning collected in the ABCD and HCP samples are displayed in Table 1. Estimates of variance explained and their confidence intervals indicated that, although effect sizes varied, EEA consistently explained a significant portion of the variance in these criterion measures (with the only exception being the relation between 0-back EEA and the flanker task in the HCP sample). In contrast, SEA explained less than 1% of the variance in each of the criterion measures and confidence intervals always contained 0. Therefore, only EEA, and not SEA, shows evidence of relations with measures of higher-order cognitive functions that have well-replicated associations with the DDM’s *v* parameter.

**Table 1:** Variance explained (*r*^*2*^) in NIH Toolbox criterion measures by efficiency of evidence accumulation (EEA) and speed of evidence accumulation (SEA) when both parameters are simultaneously included in linear mixed models and 95% confidence intervals (CIs) for *r*^2^ estimates

Sample	Measure	Task	EEA *r*^*2*^	EEA 95% CI		SEA *r*^*2*^	SEA 95% CI	
HCP	Total Cognition	0-back	0.051	*0.032*	*0.079*	0.000	*0.000*	*0.028*
		2-back	0.205	*0.164*	*0.251*	0.002	*0.000*	*0.053*
	List (working memory)	0-back	0.030	*0.014*	*0.052*	0.001	*0.000*	*0.024*
		2-back	0.091	*0.059*	*0.128*	0.003	*0.000*	*0.043*
	Card Sort (flexibility)	0-back	0.035	*0.018*	*0.062*	0.002	*0.000*	*0.030*
		2-back	0.089	*0.059*	*0.127*	0.000	*0.000*	*0.039*
	Flanker (inhibition)	0-back	0.008	*0.000*	*0.025*	0.003	*0.000*	*0.020*
		2-back	0.045	*0.025*	*0.076*	0.001	*0.000*	*0.032*
ABCD	Total Cognition	0-back	0.148	*0.135*	*0.161*	0.003	*0.000*	*0.017*
		2-back	0.202	*0.188*	*0.218*	0.006	*0.000*	*0.026*
	List (working memory)	0-back	0.079	*0.068*	*0.089*	0.002	*0.000*	*0.013*
		2-back	0.109	*0.097*	*0.120*	0.002	*0.000*	*0.014*
	Card Sort (flexibility)	0-back	0.057	*0.048*	*0.067*	0.003	*0.000*	*0.013*
		2-back	0.062	*0.053*	*0.073*	0.004	*0.000*	*0.015*
	Flanker (inhibition)	0-back	0.041	*0.033*	*0.049*	0.000	*0.000*	*0.009*
		2-back	0.060	*0.051*	*0.071*	0.001	*0.000*	*0.012*

*Note.* ABCD = Adolescent Brain Cognitive Development Study; HCP = Human Connectome Project.

Italics indicate the 95% CI values.

## Discussion

This report sought to provide greater clarity about whether the drift rate parameter (*v*) of the DDM (Ratcliff et al., 2016) can be thought of as a measure of “processing speed,” a traditional psychometric construct defined as an individual’s basic speed of information processing across nearly all cognitive tasks (Fry & Hale, 1996, 2000; Kail & Salthouse, 1994; Salthouse, 1996). We fit both the DDM and a racing accumulator model, the LBA, to data from three tasks across a set of three samples that varied substantially by age and by the participant sampling methods used. The LBA allows estimates of two dissociable features of information processing:

1. The speed of evidence accumulation (SEA), which reflects the speed with which individuals accumulate evidence, regardless of the quality of that evidence, and is therefore closely aligned with traditional construct of “processing speed”, and
2. The efficiency of evidence accumulation (EEA), which reflects individuals’ ability to selectively accumulate evidence relevant to task goals instead of task-irrelevant evidence, which has an ambiguous relationship to the traditional construct of “processing speed.”

We sought to clarify whether SEA or EEA was more strongly related to the DDM’s* v* parameter and to measures of higher-order cognitive abilities with which the *v* parameter has been previously linked.

Across all tasks and data sets, EEA was consistently strongly related (*r* >.50) to the DDM’s *v* parameter, and in nearly all tasks this relationship was strong enough that *v* could be thought of as a proxy measure for EEA (*r* >.80). In contrast, SEA displayed far weaker relations with *v* (*r* =.14–.35). When both LBA parameters were included in a multivariate regression predicting* v*, EEA consistently explained many times more variance in *v* than SEA did. Furthermore, when investigating EEA’s and SEA’s relations with measures of higher-order cognitive functions (general cognitive ability, working memory, executive functioning) that have well-replicated links to *v*, EEA consistently explained meaningful portions of the variance in all measures, whereas SEA did not explain more than 1% of the variance in any of them. Taken together, these results indicate that, from a functional standpoint in cognitive individual differences research, the DDM’s* v* parameter is primarily an index of the efficiency with which individuals selectively process relevant - as opposed to irrelevant - information, rather than an index of the overall speed with which they process information.

We used a racing accumulator modeling framework to pull apart SEA from EEA. The “processing speed” construct, as traditionally defined, would be expected to manifest in SEA, and has at best a weak and ambiguous conceptual link with EEA. In accumulator models, EEA and SEA can vary completely independently of one another and only SEA theoretically represents the overall speed with which information is processed. Although *v* and EEA can also influence the most common traditional summary measure of processing speed, mean choice RT, these relations are not as strong or selective as the relation of SEA with mean choice RT (Supplemental Materials). Thus, if widely accepted theoretical and empirical definitions of “processing speed” are taken at face value, our results, which show the DDM’s *v* parameter primarily indexes EEA and not SEA, suggest that *v* has little to do with processing speed and much more to do with individuals’ efficiency of parsing task-relevant from task-irrelevant information.

Further questions arise as to the mechanistic interpretation of SEA and EEA in terms of more basic cognitive processes. Providing a detailed discussion of these questions is beyond the scope of this report and this topic is an active area of inquiry in the field. SEA has only been explored in a limited number of studies (Hawkins & Heathcote, 2021; Miletić et al., 2021; Stevenson et al., 2022; van Maanen et al., 2016; Weigard et al., 2023), and authors have suggested interpretations of SEA in terms of “urgency” (Hawkins & Heathcote, 2021; Miletić et al., 2021) or “arousal” (van Maanen et al., 2016). EEA, in contrast, has been posited to be more closely related to the efficacy of selective attention or cognitive control processes that allow individuals to parse goal-relevant information from goal-irrelevant information (Weigard et al., 2021; Weigard & Sripada, 2021). Importantly, SEA, and EEA can each also reflect factors that are related to stimulus properties rather than individual differences, such as the intensity and discriminability of choice options (Stafford & Gurney, 2004; van Ravenzwaaij et al., 2020). Additional work is required to disentangle the multiplicity of factors that contribute SEA and EEA and elucidate the mechanistic basis of their inter-individual differences.

Although we found very strong relationships between *v* and EEA in tasks from two large community-recruited samples (ABCD and HCP), the relation between* v* and EEA was not as strong or selective in the numerosity discrimination task drawn from an online sample of Prolific participants. This finding may be due to differences in the cognitive domain involved in the Prolific study (perceptual discrimination) or due to features of Prolific participants. For example, Prolific participants may be generally higher-functioning (producing range restriction) compared with community-recruited samples in which people with lower cognitive abilities may be better represented. Nonetheless, we note that EEA continues to explain more than three times the amount of variance in *v* from the numerosity task than SEA does, suggesting that our general pattern of findings holds up well across all samples and all tasks.

Another important caveat is that our inferences are based on the assumption that the LBA and DDM provide appropriate theoretical accounts of the data. If both models fail to reflect the underlying data-generating process, their parameter estimates may nonetheless behave similarly if the models describe the data in similar (but incorrect) ways. The fact that the LBA provided very good fit across all tasks and samples (Figs. 2, 3, and 4) lends confidence to our assumption that the LBA’s EEA and SEA parameters reflect the appropriate data generating processes on these tasks. As the DDM’s *v* parameter was consistently related to EEA both in tasks in which the DDM also provided very good fit (0-back, numerosity discrimination) and in tasks in which the DDM’s fit was relatively poorer (2-back), we can infer that *v* is a fairly robust index of the EEA data-generating process across many applications of the DDM. Nonetheless, we cannot completely rule out the possibility that these tasks involve data-generating processes that are very different from those assumed by the LBA and that may be supported by future work. However, this caveat applies across essentially all cognitive model applications.

In summary, our results strongly suggest that the DDM’s *v* parameter should not be equated with the traditional construct of processing speed. This is supported by our findings that *v*, and its relations with other cognitive abilities, cannot be explained only by individuals’ speed of evidence accumulation and instead largely reflects individuals’ ability to parse relevant from irrelevant information, a mechanism that can vary independently of speed. At the very least, this pattern of results suggests that *v*’s determinants are far too complex to attribute solely to “speed of information processing.” As noted in the beginning of this report, the value of cognitive models lies in their precise formal definitions of the mechanisms underlying cognitive performance. Prior work in cognitive science has instead often relied on verbal definitions of cognitive constructs, which are inherently less precise. We acknowledge that, for the sake of communicating to broader audiences and establishing bridges with prior work, it can be tempting to map cognitive model parameters onto these popular verbally defined constructs. However, as even the highly intuitive mapping of *v* to “processing speed” appears deeply questionable, this approach may add more confusion than clarity to model-based studies. Instead, we believe that cognitive modeling research programs should rely on an ontology of processes that are defined clearly in terms of formal model parameters and their respective functional roles. Moreover, theorists should acknowledge that these formally defined constructs may resist straightforward mapping to more traditional terms and concepts.

## Supplementary Information

Below is the link to the electronic supplementary material.

Supplementary file1 (DOCX 758 KB)

## Data Availability

ABCD data are available from the NIMH Data Archive (https://nda.nih.gov/) with a valid data use agreement. HCP data are available from the HCP consortium (https://www.humanconnectome.org/study/hcp-young-adult/data-releases) after creating a HCP account. The numerosity task data will be made publicly available shortly.

## Footnotes

1 We note that, although this conceptualization of “processing speed” has been widely referenced in the literature, alternative operationalizations of “speed,” including the inspection time paradigm, may have different relations with behavioral measures and cognitive model parameters (Leite, 2009; Vickers & Smith, 1986).

2 We provide a simulation script that allows users to explore the relationship between behavioral summary statistics (mean choice RT, accuracy) and evidence-accumulation model parameters, including the *v* parameter of the DDM and SEA and EEA parameters from racing accumulator models. It shows that *v* and EEA both strongly affect accuracy (which is often taken as a proxy of quality of cognitive processing) and to a lesser degree affect mean RT. In contrast, SEA almost exclusively affects mean RT.

## Code availability

Analysis code for this study is available at: osf.io/9nd4h/?view_only=754e1f658f994d758d92ea2821153099

## References

[CR1] Ackerman, P. L., & Lohman, D. F. (2006). Individual differences in cognitive functions. In P. A. Alexander & P. H. Winne (Eds.), *Handbook of educational psychology* (pp. 139–161). Erlbaum.

[CR2] Akshoomoff, N., Beaumont, J. L., Bauer, P. J., Dikmen, S. S., Gershon, R. C., Mungas, D., ... Heaton, R. K. (2013). VIII. NIH toolbox cognition battery (CB): Composite scores of crystallized, fluid, and overall cognition. *Monographs of the Society for Research in Child Development,**78*(4), 119–132. 10.1111/mono.12038 PMC4103789 23952206

[CR3] Brown, S. D., & Heathcote, A. (2008). The simplest complete model of choice response time: Linear ballistic accumulation. *Cognitive Psychology,**57*(3), 153–178. 18243170 10.1016/j.cogpsych.2007.12.002

[CR4] Carroll, J. B. (2003). The higher-stratum structure of cognitive abilities: Current evidence supports g and about ten broad factors. In H. Nyborg (Ed.), *The scientific study of general intelligence* (pp. 5–21). Pergamon.

[CR5] Casey, B. J., Cannonier, T., Conley, M. I., Cohen, A. O., Barch, D. M., Heitzeg, M. M., … ABCD Imaging Acquisition Workgroup. (2018). The adolescent brain cognitive development (ABCD) study: Imaging acquisition across 21 sites. *Developmental Cognitive Neuroscience,**32*, 43–54. 10.1016/j.dcn.2018.03.001 PMC5999559 29567376

[CR6] Damaso, K. A., Castro, S. C., Todd, J., Strayer, D. L., Provost, A., Matzke, D., & Heathcote, A. (2021). A cognitive model of response omissions in distraction paradigms. *Memory & Cognition*. 10.3758/s13421-021-01265-z 10.3758/s13421-021-01265-z 34950999

[CR7] Donkin, C., Brown, S. D., & Heathcote, A. (2009). The overconstraint of response time models: Rethinking the scaling problem. *Psychonomic Bulletin & Review,**16*, 1129–1135. 19966267 10.3758/PBR.16.6.1129

[CR8] Donkin, C., Brown, S., Heathcote, A., & Wagenmakers, E.-J. (2011). Diffusion versus linear ballistic accumulation: Different models but the same conclusions about psychological processes? *Psychonomic Bulletin & Review,**18*, 61–69. 21327360 10.3758/s13423-010-0022-4 PMC3042112

[CR9] Dutilh, G., Annis, J., Brown, S. D., Cassey, P., Evans, N. J., Grasman, R. P., ..., & Donkin, C. (2019). The quality of response time data inference: A blinded, collaborative assessment of the validity of cognitive models. *Psychonomic Bulletin & Review*, *26*, 1051–1069. 10.3758/s13423-017-1417-2 PMC6449220 29450793

[CR10] Eisenberg, I. W., Bissett, P. G., Zeynep Enkavi, A., Li, J., MacKinnon, D. P., Marsch, L. A., & Poldrack, R. A. (2019). Uncovering the structure of self-regulation through data-driven ontology discovery. *Nature Communications,**10*(1), Article 2319. 31127115 10.1038/s41467-019-10301-1 PMC6534563

[CR11] Fry, A. F., & Hale, S. (1996). Processing speed, working memory, and fluid intelligence: Evidence for a developmental cascade. *Psychological Science,**7*(4), 237–241.

[CR12] Fry, A. F., & Hale, S. (2000). Relationships among processing speed, working memory, and fluid intelligence in children. *Biological Psychology,**54*(1/3), 1–34. 11035218 10.1016/s0301-0511(00)00051-x

[CR13] Garavan, H., Bartsch, H., Conway, K., Decastro, A., Goldstein, R., Heeringa, S., ... Zahs, D. (2018). Recruiting the ABCD sample: Design considerations and procedures. *Developmental Cognitive Neuroscience,**32*, 16–22. 10.1016/j.dcn.2018.04.004 PMC6314286 29703560

[CR14] Gelman, A., & Rubin, D. B. (1992). Inference from iterative simulation using multiple sequences. *Statistical Science,**7*(4), 457–472.

[CR15] Gelman, A., Meng, X.-L., & Stern, H. (1996). Posterior predictive assessment of model fitness via realized discrepancies. *Statistica Sinica,**6*(4), 733–760.

[CR16] Hawkins, G. E., & Heathcote, A. (2021). Racing against the clock: Evidence-based versus time-based decisions. *Psychological Review,**128*(2), 222–263. 33600202 10.1037/rev0000259

[CR17] Heathcote, A., & Matzke, D. (2022). Winner takes all! What are race models, and why and how should psychologists use them? *Current Directions in Psychological Science,**31*(5), 383–394.

[CR18] Heathcote, A., Lin, Y.-S., Reynolds, A., Strickland, L., Gretton, M., & Matzke, D. (2019). Dynamic models of choice. *Behavior Research Methods,**51*, 961–985. 29959755 10.3758/s13428-018-1067-y

[CR19] Hedge, C., Vivian-Griffiths, S., Powell, G., Bompas, A., & Sumner, P. (2019). Slow and steady? Strategic adjustments in response caution are moderately reliable and correlate across tasks. *Consciousness and Cognition,**75*, Article 102797. 31421398 10.1016/j.concog.2019.102797 PMC6920044

[CR20] Hedge, C., Powell, G., Bompas, A., & Sumner, P. (2022). Strategy and processing speed eclipse individual differences in control ability in conflict tasks. *Journal of Experimental Psychology. Learning, Memory, and Cognition,**48*(10), 1448–1469. 34591554 10.1037/xlm0001028 PMC9899369

[CR21] Kail, R., & Salthouse, T. A. (1994). Processing speed as a mental capacity. *Acta Psychologica,**86*(2/3), 199–225. 7976467 10.1016/0001-6918(94)90003-5

[CR22] Kane, M. J., & Engle, R. W. (2002). The role of prefrontal cortex in working-memory capacity, executive attention, and general fluid intelligence: An individual-differences perspective. *Psychonomic Bulletin & Review,**9*(4), 637–671. 12613671 10.3758/bf03196323

[CR23] Karalunas, S. L., & Huang-Pollock, C. L. (2013). Integrating impairments in reaction time and executive function using a diffusion model framework. *Journal of Abnormal Child Psychology,**41*(5), 837–850. 23334775 10.1007/s10802-013-9715-2 PMC3679296

[CR24] Leite, F. P. (2009). Should IQ, perceptual speed, or both be used to explain response time? *The American Journal of Psychology,**122*(4), 517–526. 20066930

[CR25] Leite, F. P., & Ratcliff, R. (2011). What cognitive processes drive response biases? A diffusion model analysis. *Judgment and Decision Making,**6*(7), 651–687.

[CR26] Lerche, V., von Krause, M., Voss, A., Frischkorn, G. T., Schubert, A.-L., & Hagemann, D. (2020). Diffusion modeling and intelligence: Drift rates show both domain-general and domain-specific relations with intelligence. *Journal of Experimental Psychology. General,**149*(12), 2207–2249. 32378959 10.1037/xge0000774

[CR27] Löffler, C., Frischkorn, G. T., Hagemann, D., Sadus, K., & Schubert, A.-L. (2024). The common factor of executive functions measures nothing but speed of information uptake. *Psychological Research*. 10.1007/s00426-023-01924-7 38372769 10.1007/s00426-023-01924-7 PMC11143038

[CR28] Matzke, D., & Wagenmakers, E.-J. (2009). Psychological interpretation of the ex-Gaussian and shifted wald parameters: A diffusion model analysis. *Psychonomic Bulletin & Review,**16*, 798–817. 19815782 10.3758/PBR.16.5.798

[CR29] McGrew, K. S. (2009). CHC theory and the human cognitive abilities project: Standing on the shoulders of the giants of psychometric intelligence research. *Intelligence,**37*(1), 1–10.

[CR30] Miletić, S., Boag, R. J., Trutti, A. C., Stevenson, N., Forstmann, B. U., & Heathcote, A. (2021). A new model of decision processing in instrumental learning tasks. *eLife,**10*, Article e63055. 33501916 10.7554/eLife.63055 PMC7880686

[CR31] Molloy, M., Lee, T., Jonides, J., Zhang, H., Sellers, J., Heathcote, A., ... Weigard, A. (in press). Joint cognitive models reveal sources of robust individual differences in conflict processing. *Computational Brain & Behavior.* 10.1007/s42113-026-00263-1 PMC13004055 41868475

[CR32] R Core Team (2013). *R: A language and environment for statistical computing*. https://cran.r-project.org/

[CR33] Ratcliff, R. (1978). A theory of memory retrieval. *Psychological Review,**85*(2), 59.

[CR34] Ratcliff, R. (2008). Modeling aging effects on two-choice tasks: Response signal and response time data. *Psychology and Aging,**23*(4), 900. 19140659 10.1037/a0013930 PMC2731573

[CR35] Ratcliff, R., Thapar, A., & McKoon, G. (2011). Effects of aging and IQ on item and associative memory. *Journal of Experimental Psychology: General,**140*(3), 464–487. 21707207 10.1037/a0023810 PMC3149731

[CR36] Ratcliff, R., Love, J., Thompson, C. A., & Opfer, J. E. (2012). Children are not like older adults: A diffusion model analysis of developmental changes in speeded responses. *Child Development,**83*(1), 367–381. 22188547 10.1111/j.1467-8624.2011.01683.x PMC3267006

[CR37] Ratcliff, R., Smith, P. L., Brown, S. D., & McKoon, G. (2016). Diffusion decision model: Current issues and history. *Trends in Cognitive Sciences,**20*(4), 260–281. 26952739 10.1016/j.tics.2016.01.007 PMC4928591

[CR38] Reinhartz, A., Strobach, T., Jacobsen, T., & von Bastian, C. C. (2023). Mechanisms of training-related change in processing speed: A drift-diffusion model approach. *Journal of Cognition,**6*(1), Article 46. 37600217 10.5334/joc.310 PMC10437139

[CR39] Salthouse, T. A. (1996). The processing-speed theory of adult age differences in cognition. *Psychological Review,**103*(3), 403–428. 8759042 10.1037/0033-295x.103.3.403

[CR40] Schmiedek, F., Oberauer, K., Wilhelm, O., Süß, H.-M., & Wittmann, W. W. (2007). Individual differences in components of reaction time distributions and their relations to working memory and intelligence. *Journal of Experimental Psychology: General,**136*(3), 414–429. 17696691 10.1037/0096-3445.136.3.414

[CR41] Schmitz, F., & Wilhelm, O. (2016). Modeling mental speed: Decomposing response time distributions in elementary cognitive tasks and correlations with working memory capacity and fluid intelligence. *Journal of Intelligence,**4*(4), Article 13.

[CR42] Schubert, A.-L., & Frischkorn, G. T. (2020). Neurocognitive psychometrics of intelligence: How measurement advancements unveiled the role of mental speed in intelligence differences. *Current Directions in Psychological Science,**29*(2), 140–146.

[CR43] Schubert, A.-L., Hagemann, D., Voss, A., Schankin, A., & Bergmann, K. (2015). Decomposing the relationship between mental speed and mental abilities. *Intelligence,**51*, 28–46.

[CR44] Schubert, A.-L., Frischkorn, G. T., Hagemann, D., & Voss, A. (2016). Trait characteristics of diffusion model parameters. *Journal of Intelligence,**4*(3), Article 7.

[CR45] Stafford, T., & Gurney, K. N. (2004). The role of response mechanisms in determining reaction time performance: Piéron’s law revisited. *Psychonomic Bulletin & Review,**11*, 975–987. 15875968 10.3758/bf03196729

[CR46] Starns, J. J., Cataldo, A. M., Rotello, C. M., Annis, J., Aschenbrenner, A., Bröder, A., … Wilson, J. (2019). Assessing theoretical conclusions with blinded inference to investigate a potential inference crisis. *Advances in Methods and Practices in Psychological Science,**2*(4), 335–349.

[CR47] Stevenson, N., Innes, R., Miletic, S., & Boag, R. J. (2024). Joint modelling of latent cognitive mechanisms shared across decision-making domains. *Computational Brain & Behavior,**7*(1), 1–22. 38425991 10.1007/s42113-023-00192-3 PMC10899373

[CR48] Thurstone, L. L. (1931). Multiple factor analysis. *Psychological Review,**38*(5), 406–427.

[CR49] Turner, B. M., Sederberg, P. B., Brown, S. D., & Steyvers, M. (2013). A method for efficiently sampling from distributions with correlated dimensions. *Psychological Methods,**18*(3), 368–384. 23646991 10.1037/a0032222 PMC4140408

[CR50] Usher, M., & McClelland, J. L. (2001). The time course of perceptual choice: The leaky, competing accumulator model. *Psychological Review,**108*(3), 550–592. 11488378 10.1037/0033-295x.108.3.550

[CR51] Van Essen, D. C., Smith, S. M., Barch, D. M., Behrens, T. E., Yacoub, E., Ugurbil, K., WU-Minn HCP Consortium. (2013). The WU-Minn human connectome project: An overview. *Neuroimage,**80*, 62–79. 23684880 10.1016/j.neuroimage.2013.05.041 PMC3724347

[CR52] van Maanen, L., Forstmann, B. U., Keuken, M. C., Wagenmakers, E.-J., & Heathcote, A. (2016). The impact of MRI scanner environment on perceptual decision-making. *Behavior Research Methods,**48*(1), 184–200. 25701105 10.3758/s13428-015-0563-6 PMC4819926

[CR53] van Ravenzwaaij, D., Brown, S., & Wagenmakers, E.-J. (2011). An integrated perspective on the relation between response speed and intelligence. *Cognition,**119*(3), 381–393. 21420077 10.1016/j.cognition.2011.02.002

[CR54] van Ravenzwaaij, D., Brown, S. D., Marley, A., & Heathcote, A. (2020). Accumulating advantages: A new conceptualization of rapid multiple choice. *Psychological Review,**127*(2), 186–215. 31580104 10.1037/rev0000166

[CR55] Vermeent, S., Young, E. S., DeJoseph, M. L., Schubert, A.-L., & Frankenhuis, W. E. (2024). Cognitive deficits and enhancements in youth from adverse conditions: An integrative assessment using Drift Diffusion Modeling in the ABCD study. *Developmental Science,**27*, Article e13478. 38321588 10.1111/desc.13478 PMC11338291

[CR56] Vermeent, S., Young, E. S., Van Gelder, J.-L., & Frankenhuis, W. E. (2024). Childhood adversity is not associated with lowered inhibition, but lower perceptual processing: A drift diffusion model analysis. *Cognitive Development,**71*, Article 101479.

[CR57] Vickers, D., & Smith, P. L. (1986). The rationale for the inspection time index. *Personality and Individual Differences,**7*(5), 609–623.

[CR58] Voss, A., Rothermund, K., & Voss, J. (2004). Interpreting the parameters of the diffusion model: An empirical validation. *Memory & Cognition,**32*(7), 1206–1220. 15813501 10.3758/bf03196893

[CR59] Weigard, A., & Huang-Pollock, C. (2017). The role of speed in ADHD-related working memory deficits: A time-based resource-sharing and diffusion model account. *Clinical Psychological Science,**5*(2), 195–211. 28533945 10.1177/2167702616668320 PMC5437983

[CR60] Weigard, A., & Sripada, C. (2021). Task-general efficiency of evidence accumulation as a computationally defined neurocognitive trait: Implications for clinical neuroscience. *Biological Psychiatry: Global Open Science,**1*(1), 5–15. 35317408 10.1016/j.bpsgos.2021.02.001 PMC8936715

[CR61] Weigard, A., Huang-Pollock, C., Brown, S., & Heathcote, A. (2018). Testing formal predictions of neuroscientific theories of ADHD with a cognitive model–based approach. *Journal of Abnormal Psychology,**127*(5), 529. 30010369 10.1037/abn0000357 PMC6091877

[CR62] Weigard, A., Clark, D. A., & Sripada, C. (2021). Cognitive efficiency beats top-down control as a reliable individual difference dimension relevant to self-control. *Cognition,**215*, Article 104818. 34252724 10.1016/j.cognition.2021.104818 PMC8378481

[CR63] Weigard, A., Matzke, D., Tanis, C., & Heathcote, A. (2023). A cognitive process modeling framework for the ABCD study stop-signal task. *Developmental Cognitive Neuroscience,**59*, Article 101191. 36603413 10.1016/j.dcn.2022.101191 PMC9826813

[CR64] Weigard, A., Angstadt, M., Taxali, A., Heathcote, A., Heitzeg, M. M., & Sripada, C. (2024a). Flexible adaptation of task-positive brain networks predicts efficiency of evidence accumulation. *Communications Biology,**7*(1), 801. 38956310 10.1038/s42003-024-06506-w PMC11220037

[CR65] Weigard, A., Suzuki, T., Skalaban, L. J., Conley, M., Cohen, A. O., Garavan, H., ... Heathcote, A. (2024b). Dissociable contributions of goal-relevant evidence and goal-irrelevant familiarity to individual and developmental differences in conflict recognition. *Cognitive Science,**48*(11), Article e70019. 10.1111/cogs.70019 PMC11589665 39587984

[CR66] Weintraub, S., Dikmen, S. S., Heaton, R. K., Tulsky, D. S., Zelazo, P. D., Bauer, P. J., & Wallner-Allen, K. (2013). Cognition assessment using the NIH toolbox. *Neurology,**80*(11 Supplement 3), S54–S64. 23479546 10.1212/WNL.0b013e3182872ded PMC3662346

[CR67] WU-Minn Consortium (2017). *WU-Minn HCP 1200 subjects data release reference manual. *Human Connectome Project*. *https://www.humanconnectome.org

