# Supplementary material for: The diffusion model’s drift rate parameter primarily reflects efficiency, rather than speed, of evidence accumulation
Source: Psychon Bull Rev. 2026 Feb 26;33(3):100. doi: 10.3758/s13423-026-02861-3 (PMC12945898; doi:10.3758/s13423-026-02861-3)
Supplement: Supplementary file 1 — Supplementary file1 (DOCX 758 KB) [file 13423_2026_2861_MOESM1_ESM.docx]

**Supplemental Materials**

**EEA’s and SEA’s Relations with Summary Statistics**

Given the association of the traditional conceptualization of “processing speed” with measures of mean response time (RT), we used simulations to investigate the impact of alternately varying EEA and SEA on both mean RT and accuracy rates. Code available in our OSF page (“EEA_SEA_effects_sim.R” at osf.io/9nd4h) demonstrates across the linear ballistic accumulator and racing diffusion models that EEA has a substantial impact on accuracy and modest impact on mean RT, similar to the impacts of the DDM’s *v* parameter on observed behavior (Matzke & Wagenmakers, 2009). In contrast, SEA has a substantial impact on mean RT and a minimal impact on accuracy. This is illustrated by the simulations displayed in Supplemental Figure 1, which involved simulating data (20,000 trials per data set) from LBA models in which EEA and SEA were systematically varied between the values of 0 and 4 while holding the value of the other process at a constant of 2. EEA variation causes accuracy to vary over a wide range from chance accuracy to 80% while variation in SEA only causes a few percentage points of change at most. Higher EEA increases the speed of correct RTs and decreases the speed of error RTs, the effects of which balance out to create a negligible net effect on the overall average of RTs. In contrast, higher SEA causes a dramatic decrease in both correct and error RTs. Therefore, as the traditional construct of “processing speed” was primarily measured using mean RTs, we can assume that the SEA parameter has a stronger conceptual and methodological link to this construct than the EEA parameter does.

**Comparisons of Threshold and Nondecision Parameters**

In addition to our primary analyses focused on relations between the DDM’s *v* parameter and estimates of EEA and SEA from the DDM, we also sought to determine whether the DDM and LBA provide similar estimates of other parameters that have analogous interpretations across models. We estimated the height of the response thresholds for all LBA model fits by adding the parameter for the top of the start point distribution (*A*) to the parameter for the height of threshold above *A* (*B*) and then averaging the resulting threshold height value (*b = A + B*) across all task conditions. We then investigated correlations of these threshold height estimates with the DDM’s response threshold separation (*a*) parameter, which is thought to similarly index individuals’ level of response caution. Next, we investigated correlations between the mean nondecision time parameters estimated in the LBA and DDM. Scatterplots and Pearson correlation values for all cross-model associations are displayed in Supplemental Figure 2. Consistent with prior work (Donkin et al., 2011), correlations for these analogous parameters across models were far from perfect, but were uniformly strong and positive. The correspondence was generally stronger for the n-back tasks than for the numerosity discrimination task, mirroring the main findings on EEA.

**Supplemental Tables and Figures**

| Parameter | Bounds | Mean (µ) | Scale (σ) |
| --- | --- | --- | --- |
| Boundary separation (*a*) | 0, ∞ | 1.000 | 0.500 |
| Drift rate (*v*) | -∞, ∞ | 3.000 | 1.000 |
| Starting point (*z*) | 0, 1 | 0.500 | 0.100 |
| Nondecision time (*t0*) | 0, 2 | 0.300 | 0.100 |
| Nondecision time variability (*st0*) | 0, 2 | 0.100 | 0.050 |
| “go” failure (*p_gf_*) | -∞, ∞ | 0.000 | 1.000 |

**Supplemental Table 1.** Prior distributions for diffusion decision model (DDM) parameters. Priors were truncated normal distributions defined by mean (µ) and scale (σ) parameters and the reported bounds. These broad and uninformative prior distributions were used across all tasks. Priors for the drift rate (*v*) parameter were kept constant across all experimental conditions. The “go failure” parameter (*p_gf_*) was estimated on the probit scale and the probit scale prior distribution shown in the table corresponds to a uniform distribution bounded at 0 and 1 on the probability scale.

| Parameter | Bounds | Mean (µ) | Scale (σ) |
| --- | --- | --- | --- |
| Threshold (*B*) | 0, ∞ | 1.000 | 1.000 |
| Start point variability (*A*) | 0, ∞ | 1.000 | 1.000 |
| Drift rate for matching responses (*v_match_*) | -∞, ∞ | 2.000 | 1.000 |
| Drift rate for matching responses (*v_mismatch_*) | -∞, ∞ | 1.000 | 1.000 |
| Drift rate variability (*sv*) | 0, ∞ | 1.000 | 1.000 |
| Nondecision time (*t0*) | 0.1,2 | 0.300 | 0.100 |
| “go” failure (*p_gf_*) | -∞, ∞ | 0.000 | 1.000 |

**Supplemental Table 2.** Prior distributions for linear ballistic accumulator (LBA) parameters. Priors were truncated normal distributions defined by mean (µ) and scale (σ) parameters and the reported bounds. These broad and uninformative prior distributions were used across all tasks. Priors for the drift rate (*v_match_, v_mismatch_*) parameters were kept constant across all experimental conditions. The “go failure” parameter (*p_gf_*) was estimated on the probit scale and the probit scale prior distribution shown in the table corresponds to a uniform distribution bounded at 0 and 1 on the probability scale.

**
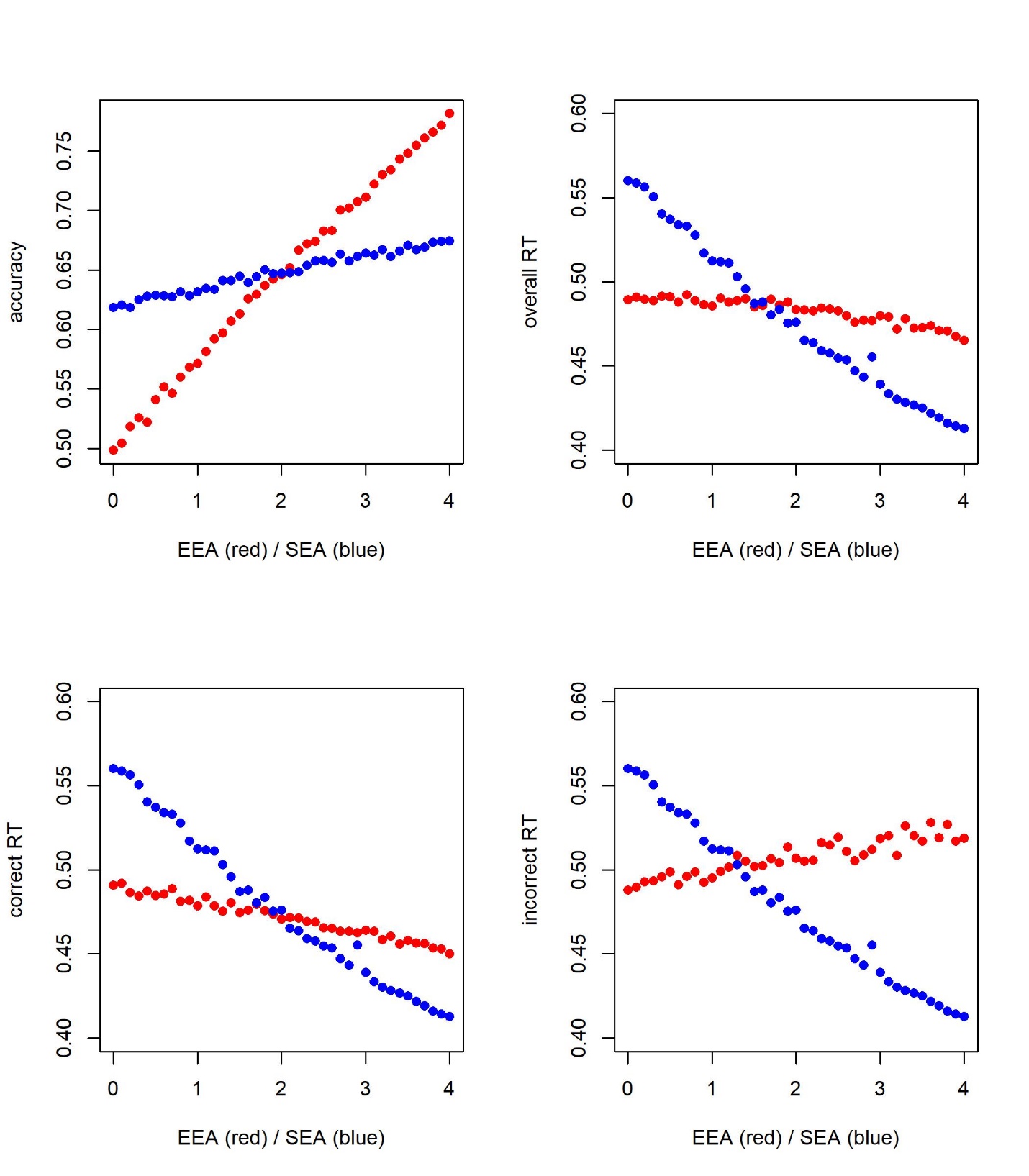
**

**Supplemental Figure 1**. Accuracy rates and mean response time (RT) for data sets simulated from LBA models that systematically vary efficiency of evidence accumulation (EEA; red) and speed of evidence accumulation (SEA; blue) between the values of 0 and 4.

**
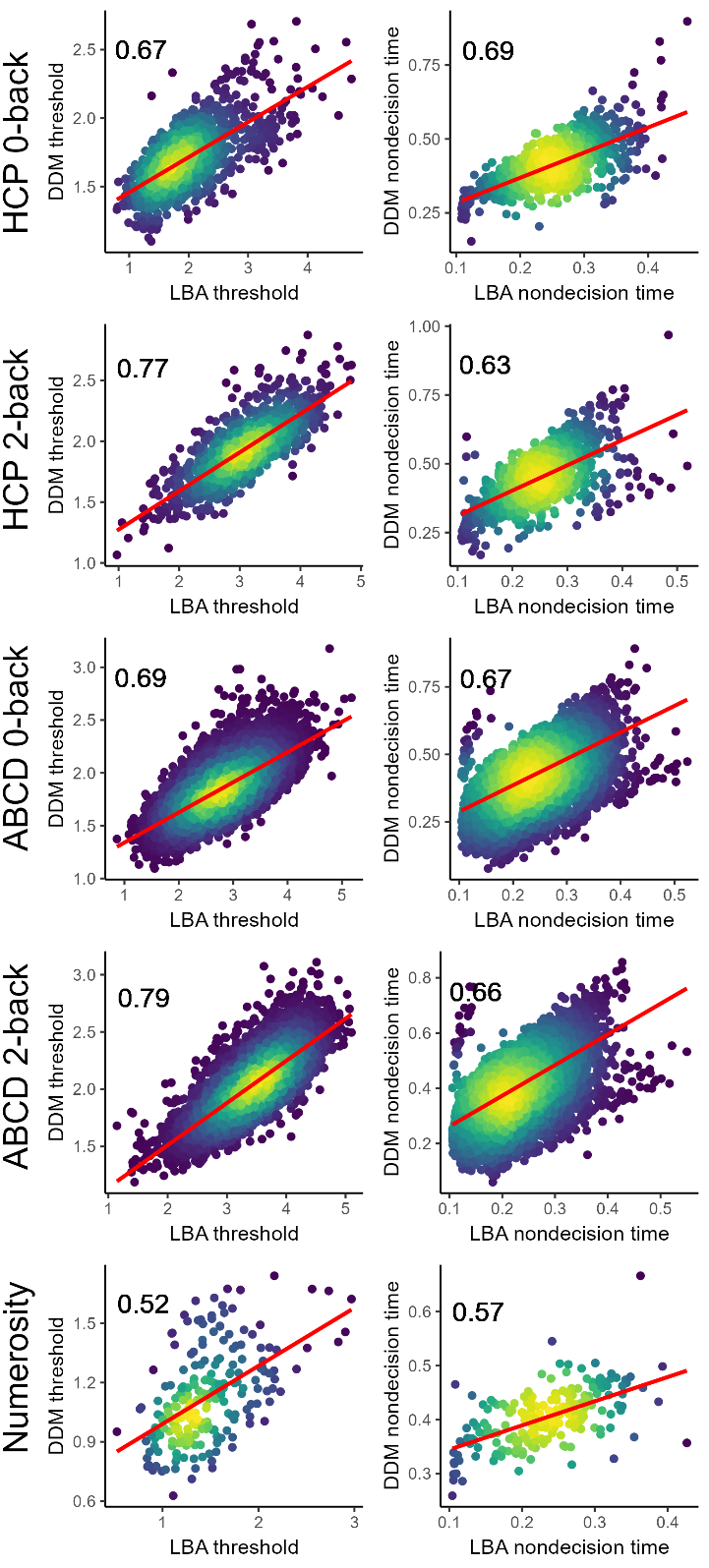
**

**Supplemental Figure 2**. Relations of response threshold and nondecision time parameters estimated across the linear ballistic accumulator model (LBA) and diffusion decision model (DDM). Rows represent individual cognitive tasks drawn from the Human Connectome Project (HCP), Adolescent Brain Cognitive Development study (ABCD), and a sample of Prolific participants who completed a numerosity discrimination task online. Pearson correlation estimates are reported in the upper left corner. The color scale reflects the density of the points, with blue indicating lower density and yellow indicting greater density, and the red lines indicate the linear relations between the variables.
